# Snake Scanning for SEM: Quantification and Correction of Its Inherent Misalignment Distortion Using an External Scan Controller

**DOI:** 10.3390/ma19010016

**Published:** 2025-12-19

**Authors:** Jieping Ding, Ling’en Liu, Ni Wang, Yixu Zhang, Liang Tang, Junxia Lu, Yuefei Zhang, Ze Zhang

**Affiliations:** 1 School of Materials Science and Engineering, Beijing University of Technology, Beijing 100124, China; 2 School of Mechanical and Electrical Engineering, Guilin University of Electronic Technology, Guilin 541004, China; 3 School of Materials Science and Engineering, Zhejiang University, Hangzhou 310058, China

**Keywords:** scanning electron microscopy, image distortion, quantitative correction, scanning strategy

## Abstract

Distortions in scanning electron microscope (SEM) images compromise characterization accuracy and restrict reliable quantitative analysis. Quantifying and correcting these distortions remains challenging due to the complexity of their inherent sources, such as scanning coil hysteresis and electronic circuit response delays. To address this, we independently developed a scanning controller and software system that enables customizable scanning strategies and is crucial for capturing unprocessed raw data. We utilized the characteristic row misalignment of snake scanning to split images into sub-images, measure offsets using the ORB algorithm, and apply pixel compensation. Experimental validation shows that corrected images exhibit reduced distortion artifacts, with structural similarity comparable to raster scanning results and improved reference-free quality metrics. The distortion magnitude is independent of magnification, primarily governed by dwell time, and stabilizes at a minimum level when the dwell time reaches a critical threshold. This work clarifies the relationship between scanning parameters and distortion behavior, guiding the optimization of SEM scanning strategies. Furthermore, it offers a potential scalable framework for distortion correction in related microscopy techniques. Many of these techniques also face distortion issues from hardware hysteresis or circuit delays, similar to SEM.

## 1. Introduction

The scanning electron microscope (SEM) is critical for high-resolution characterization in scientific research and industry, enabling applications such as in situ observation and three-dimensional surface measurement [1,2,3,4]. However, image distortion significantly degrades microscopic material characterization in practical research and engineering [5,6,7]. For instance, in semiconductor-integrated circuit inspection, it can lead to errors in determining defect size and location, thereby impairing material performance assessment. They typically manifest as time-dependent image drift and spatial distortion [8,9,10,11]. Non-inherent distortions are complex and influenced by environmental factors such as temperature and operating conditions. In contrast, inherent distortion stems from the internal hardware of the SEM system. This includes electron beam positioning errors caused by electromagnetic hysteresis in the scanning coils and delays in circuit response.

Image distortion correction primarily relies on model-based postprocessing methods, including optical distortion correction [12,13,14,15], multi-instrument complementary techniques [16,17,18], and data-driven methods [19,20,21,22]. These methods generally focus on refining acquired data rather than addressing distortion at the source of imaging signal generation. To tackle this issue, optimizing scanning strategies has emerged as a promising complementary approach. This strategy focuses on addressing distortion at the stage of beam scanning itself, aiming to prevent errors from accumulating or spreading. Lenthe WC et al. [16] systematically investigated the impact of scanning parameters, such as dwell time and deflector type, on dynamic response errors in SEM. They utilized a high-speed 16-bit digital/analog IO controller (National Instruments NI-DAQ 6363) in conjunction with the built-in scanning controller of the FEI microscope to conduct multiple sets of image acquisition and analysis experiments. The research results indicate that hardware characteristics and scanning patterns directly determine imaging stability. Although their work clarified the role of system latency in transient artifacts, it did not specifically address the inherent spatial distortion unique to snake scanning trajectories. Velazco A et al. [23] compared raster, snake, and Hilbert scan patterns, showing that snake scanning mitigates flyback-induced artifacts by eliminating abrupt beam retrace movements in scanning transmission electron microscopy (STEM). However, their focus remained on drift-related distortions in atomic-resolution lattice imaging, leaving system-inherent distortion in SEM largely unexamined. Expanding on non-traditional scanning approaches, Sang X et al. [24] developed spiral scan trajectories in STEM, illustrating that continuous, smooth beam paths enhance dose uniformity and reduce beam damage. Nevertheless, their work concentrated on high-speed imaging optimization rather than addressing intrinsic distortion mechanisms in SEM systems.

Despite these advancements, significant gaps persist in the application of snake scanning for SEM distortion correction. First, the distinctive odd–even row misalignment inherent to snake scanning—a direct indicator of system-inherent distortion—has not been utilized for quantitative measurement. Second, the relationship between dwell time and the magnitude of such distortion remains poorly characterized, thereby limiting practical guidance for parameter optimization. Finally, existing correction frameworks lack signal-level compensation strategies specifically designed to address the artifacts introduced by snake scanning.

This study integrated a self-developed external scan controller and a software system with the SEM. We utilized the snake scanning strategy to capture the original image data and perform quantification and correction of inherent image distortions. We aimed to (i) accurately quantify and correct inherent distortions by leveraging the unique odd–even row misalignment characteristic of snake scanning; (ii) carry out a comparative analysis of the imaging principles, distortion forms, and causes of raster scanning and snake scanning, providing references and guidance for the development of SEM scanning strategies in the future; (iii) provide a novel distortion quantification and correction method adaptable to other microscopy fields, such as STEM and Atomic Force Microscopy, where similar distortion challenges exist.

## 2. Methods

### 2.1. Overview

The present study focused on quantifying inherent image distortions and implementing compensation during image acquisition by using the distortion characteristics of the snake scanning strategy. First, we analyzed the causes of inherent distortion, emphasizing the impacts resulting from errors in the scanning coil system (Section 2.2). Second, we utilized the self-developed external scan controller and a software system to acquire a large number of images in both raster and snake scanning modes, conducting a comparative analysis of their imaging principles, distortion causes, and patterns (Section 2.3). Then, we measured and compensated for distortions based on the characteristics of odd–even row misalignment in snake scanning images (Section 2.4). Finally, we evaluated the quality of the corrected images and discussed the effectiveness of the distortion quantification and correction (Section 2.5).

### 2.2. Imaging Principles and Image Distortion

**Figure 1:**
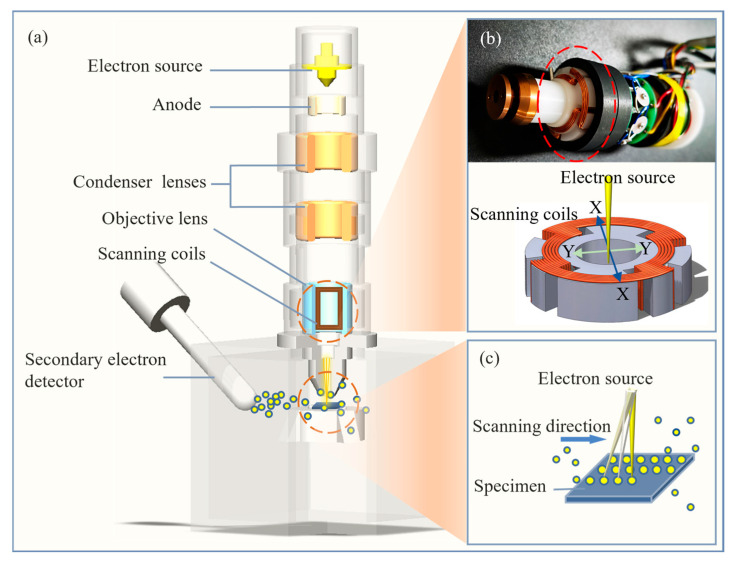
Main components and imaging principles of SEM. (**a**) The process of secondary electron imaging: the dashed circle marks two key focus areas linked to (**b,c**); (**b**) the physical and schematic diagram of the scanning coils: the red dashed circle highlights this core component, and X/Y arrows indicate electron beam deflection directions controlled by the coils; (**c**) the schematic diagram of secondary electrons (yellow circles) generated by the electron beam scanning of the sample surface.

In SEM secondary electron imaging, an electron beam is generated, accelerated by an electric field, and focused via an electromagnetic lens (Figure 1a). Scanning coils then steer the beam across the sample line by line, exciting secondary electron emission. These electrons are detected, and the resulting signals are processed to form high-resolution images. Two sets of coils deflect the beam in the X and Y-directions using time-varying currents that produce controlled magnetic fields (Figure 1b). This enables precise point-by-point scanning of the sample surface (Figure 1c).

Precise deflection control of the scanning coils is critical in the SEM imaging mechanism [25]. The deflection of the electron beam is controlled by the magnetic field of the scanning coil, and the magnetic field characteristics directly depend on the driving current of the coil. Since the scanning coil is a typical inductive element, the change in magnetic flux is inevitably lagging behind the change in the driving current [26,27]. Specifically, when the driving current changes, the coil generates an opposing electromotive force to hinder the current’s transient change. This prevents the actual current from following the ideal driving signal in real time, which in turn causes a delay in the magnetic field’s strength and distribution. Ultimately, this leads to deviations in the electron beam trajectory [28,29], as well as reduced resolution and distorted imaging quality.

### 2.3. Experimental Techniques

Theoretically, the acquired SEM images should exhibit significant distortion during each retrace period of the electron beam [14]. However, we optimized images through postprocessing to reduce distortion, which may obscure the true characteristics of inherent distortions. Commercial controllers can meet the routine imaging requirements, but they have limitations such as parameter flexibility in terms of distortion quantification and precise calibration [16]. Therefore, this study quantifies these inherent distortions using raw image data that has not been cropped or processed, thus facilitating the accurate implementation of targeted corrections.

**Figure 2:**
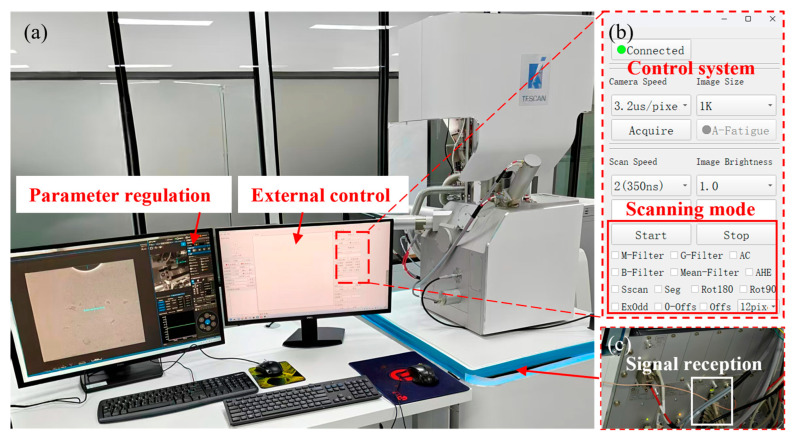
The experimental platform. (**a**) The collaborative operation of SEM and external control; (**b**) the self-developed software system; (**c**) the signal reception of an image acquisition controller.

As shown in Figure 2, the SEM images were acquired using the self-developed external scan controller and a software system. The specific design modules can be seen in Figures S1 and S2 of the Supplementary Materials. We can use the custom scanning code to freely modify the scanning strategy, retention time, and other parameters. The self-developed scanning generator is implemented based on an FPGA to achieve real-time synchronization of scanning waveform generation and signal acquisition, and supports the output of raw data without preprocessing. The experiment was conducted on a Tescan-Mira4 SEM (Tescan s.r.o., Brno, Czech Republic). A secondary electron detector was used to collect signals, with the acceleration voltage set to 20 keV and the beam current at 3 nA. We selected the AGS1937 Universal Tin on Carbon specimen (Agar Scientific Ltd., Stansted, UK) and second-generation nickel-based single crystal superalloy for experimental verification.

**Figure 3:**
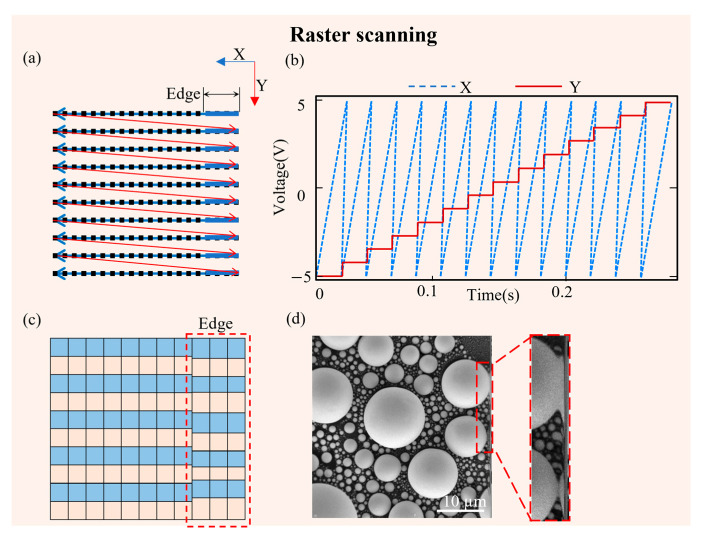
Schematic diagram of raster scanning. (**a**) The electron beam moves point by point (the small black square) along the directions indicated by the red and blue arrows; (**b**) the voltage applied to the X and Y scanning coils during a single frame acquisition; (**c**) the odd-rows (in blue) of the image pixels are arranged together with the even-rows (in pink), and the distortion at the image edges (indicated by the red dashed box); (**d**) the image edge distortion can be observed in the local magnification diagram.

Existing commercial SEMs typically employ raster scanning to acquire images. To facilitate distinction during the image acquisition process, we have designed an external scan controller to scan from right to left (commercial SEMs usually scan from left to right), without affecting the overall signal reception. Once a line is completed, the beam quickly returns to the starting position of the next line to begin scanning again (Figure 3a). We defined the X-direction as the row direction and the Y-direction as the column direction (Figure 3b). For scanning at the micrometer level, the typical voltage range can cover ±4 V to ±6 V. We adjusted the voltage range of ±5 V according to the field of view size of the SEM used in the experiment. In the raster scanning method, the relationship between the row direction voltage and time presents a sawtooth waveform, characterized by a slow rise followed by a sharp drop. The column direction is increasing row by row. In the unprocessed original image, we can observe irregular distortions occurring on the right side of the image (at the starting point of the electron beam scan), such as stretching or twisting (Figure 3c,d).

**Figure 4:**
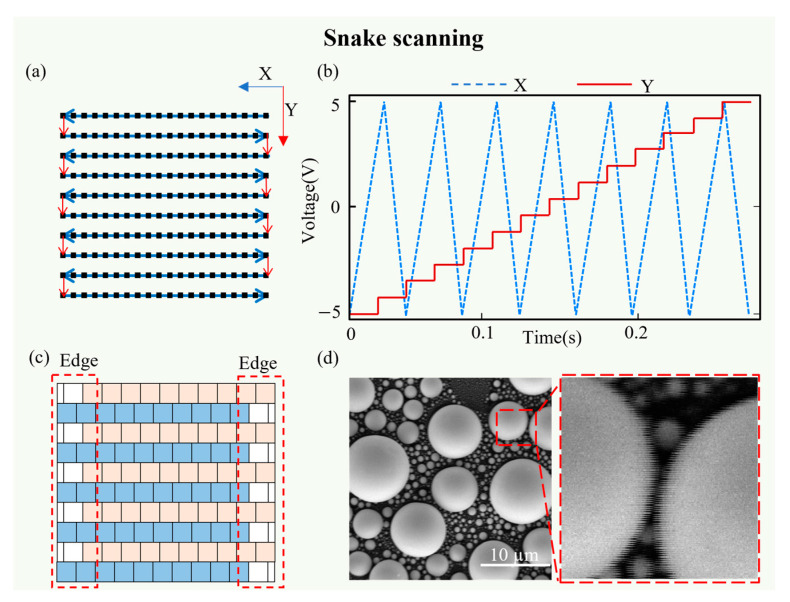
Schematic diagram of snake scanning. (**a**) The electron beam moves point by point (the small black square) along the directions indicated by the red and blue arrows; (**b**) the voltage applied to the X and Y scanning coils during a single frame acquisition; (**c**) the odd-rows (in blue) of the image pixels are arranged out of alignment with the even-rows (in pink) together, and the pixels that require compensation for image edges (in white) are shown within the red dashed box; (**d**) actual SEM images from snake scanning are shown in the magnified local images.

Meanwhile, the electron beam scans point by point from right to left during the snake scanning process. After completing one row, it shifts vertically to the starting position of the next scanning line (i.e., the left side of the image), following a continuous, end-to-end movement pattern (Figure 4a). Consequently, the voltage–time relationship manifests of X-direction as a symmetrical triangular waveform (Figure 4b). Different from the sawtooth wave of raster scanning, the triangular wave reflected the continuous movement of the electron beam within each scanning cycle without the pause of the flyback. In addition, the triangular wave induces a ramp response, and the steady-state hysteresis effect results in a quantifiable global offset in the image. Current direction reversal between odd and even rows causes misalignment in the scanning trajectory. This results in ghosting or misalignment distortions in the image (Figure 4c,d). Comparing the distortion patterns of the two scanning methods, raster scanning results in irregular distortions concentrated at one edge of the image. In contrast, snake scanning produced a regular odd–even row misalignment distortion across the entire image.

### 2.4. Measurement and Compensation of Image Distortion

#### 2.4.1. Feature Points Extraction and Matching

There are significant differences between raster scanning and snake scanning in imaging mechanisms and distortion modes. This section focuses on the odd–even row misalignment distortion commonly encountered in snake scanning, establishing it as a basis for quantifying distortions. The images acquired in raster scanning mode served as the control group, while those acquired in snake scanning mode were designated as the measurement and compensation group. We adopted identical imaging settings for both scanning modes, including magnification, dwell time, acceleration voltage, working distance, and image brightness and contrast.

**Figure 5:**
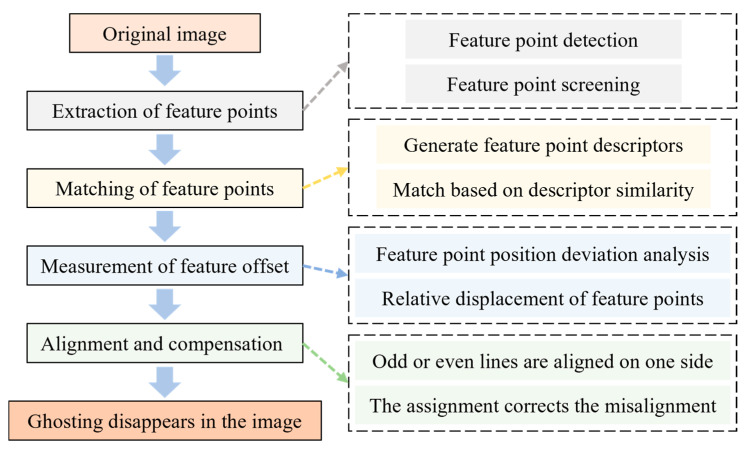
Flow chart of measurement and compensation of distortion.

Feature matching and signal compensation are classic and widely used techniques in image analysis and signal processing [30]. We utilized the OpenCV library within the Python (version 3.9) platform and adopted the Oriented FAST and Rotated BRIEF (ORB) algorithm for feature matching [30,31]. The traditional cross-correlation method, as a registration technique based on grayscale intensity, is highly sensitive to noise. The ORB algorithm can achieve more stable feature point matching in experiments conducted under short dwell times. Then, we aligned one side of the odd and even rows and compensated for the signal loss on the other side using an assignment method. The overall process involved several key steps: feature point extraction, feature point matching, error calculation, alignment, and compensation, as shown in Figure 5.

When extracting feature points with the ORB algorithm, we first detect stable feature points, achieve scale invariance via an image pyramid (detecting FAST corners at different resolutions), and rotation invariance via the intensity centroid method (calculating orientations for invariant descriptors) to avoid geometric transformation-induced matching errors. We adopted an approximate nearest neighbor matcher based on the Fast Library for Approximate Nearest Neighbors (FLANN) algorithm [32,33] to match the feature points in the two sub-images. The FLANN approach quickly identifies the approximate nearest neighbors of a given point in high-dimensional space. We then applied K-nearest neighbor matching (with k = 2) to find the two nearest neighbors of the feature points. The Euclidean distance was used to measure the similarity between the feature points and screen the best matching points according to the distance. The coordinates of the feature points were extracted from the original and target images based on the filtered matching points. We then measure feature offsets using image coordinates to calculate odd–even row pixel offsets, incorporate these into a model to quantify displacement, and finally align/compensate odd–even rows, with displacement differences indicating image distortion.

**Figure 6:**
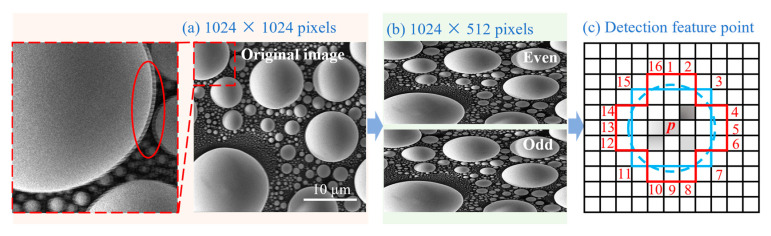
Schematic diagram of feature matching after splitting the even and odd rows of the image. (**a**) The original image with a size of 1024 × 1024 pixels, and the partial magnified view (oval shape) of the red dashed line on the left shows the misalignment of odd and even rows; (**b**) split into two sub-images of 1024 × 512 pixels; (**c**) the schematic diagram of the feature point detector is based on the ORB algorithm: the red rectangular frame delimits the core local neighborhood of the candidate feature point, and the blue closed curve (circle) encloses 16 surrounding pixels that serve as the detection sample set in the FAST corner detection criterion.

During the image acquisition stage of the snake-shaped scanning, a strategy of receiving data after separating the odd rows and even rows was adopted. As shown in Figure 6a, the original image, with a size of 1024 × 1024 pixels, exhibited distortion by ghosting. We preprocessed the image by precisely splitting it into two sub-images, with a size of 1024 × 512 pixels, based on odd and even rows (Figure 6b). Subsequently, we used the ORB algorithm on the Python platform to create a feature detector (Figure 6c), setting the threshold for the number of detected feature points to 5000. We then detected feature points and computed descriptors for the sub-images.

To clarify the distortion compensation direction, we calculated and analyzed X and Y offset ranges before the distortion measurement experiment. We collected images with dwell times of 350 ns, 760 ns, 1.1 μs, 3.2 μs, and 8.8 μs, as well as magnifications of 3kx, 4kx, 5kx, 6kx, 10kx, and 20kx. The scope of collection includes both rapid scanning and slow scanning, as well as high and low magnification rates. As shown in Table 1, the numerical values represent the offset amounts, wherein a negative sign indicates the direction of the offset. The offset in the X-direction fluctuated between 3 and 11. The offset in the Y-direction was mostly 0 and occasionally showed an offset of −1. The offset in the X-direction dominates and significantly impacts image distortion. In contrast, the offset in the Y-direction was relatively minor, typically less than 1 pixel. This pattern of offset differences can be explained by the way the voltages change in the X and Y directions. The voltage in the X-direction continuously changed, while the voltage in the Y-direction increased only in a stepwise manner (Figure 3b and Figure 4b). The minimum compensation unit was set as 1 pixel based on the established compensation rules. Consequently, the subsequent work centered around the measurement and compensation of the offset in the X-direction.

#### 2.4.2. Compensation Process and Impact

**Figure 7:**
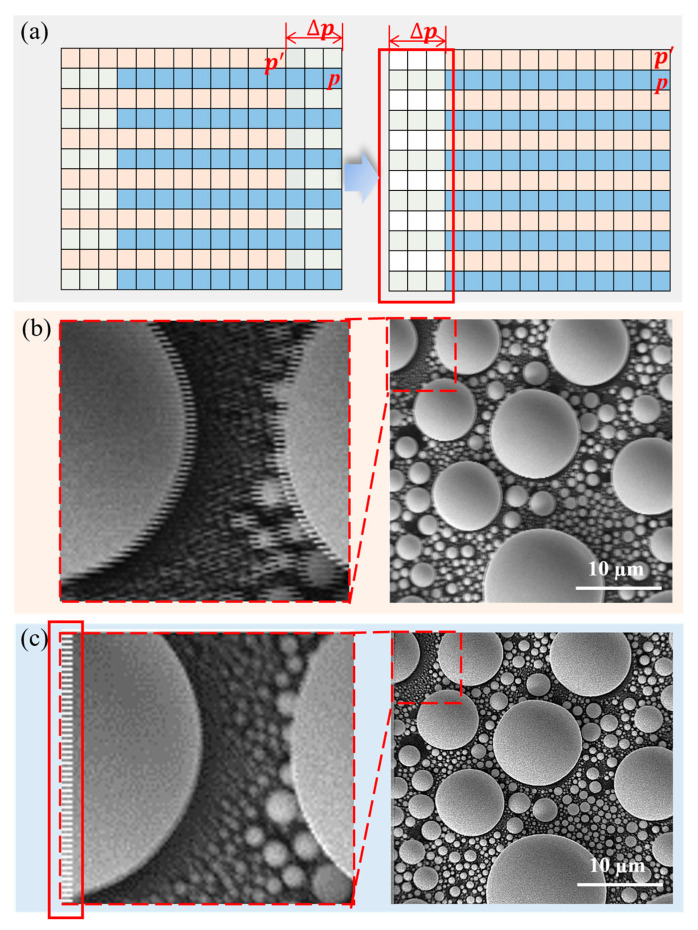
Schematic diagram of compensation. (**a**) The pink and blue sections represent the odd and even rows of image pixels respectively, while the green and white (the red solid line box) represent the required pixel compensation amounts for them; (**b**) the image misalignment before pixel compensation (partial magnified image of the red dashed line); (**c**) the image after pixel compensation, and the red solid line frame on the left corresponds to (**a**).

The schematic diagram of the compensation process is shown in Figure 7a. The pixel position p of the feature points in the even-row sub-image served as the reference. The corresponding pixel position $p^{\prime }$ in the odd-row sub-image is shifted toward the reference position by the distortion amount ∆p. Taking the even-row sub-image as the reference, the entire odd-row sub-image was shifted to the right, resulting in missing pixels on its left side (Figure 7a). We compensated for the missing pixels by setting their pixel values to 65,535 (pixel maximum value, displayed in white) to visualize the pixel offset. Figure 7b,c shows the original and compensated image, respectively. In the magnified view of Figure 7b, ghosting was visible in the original image. After compensation (Figure 7c), it was clear that the ghosting had been eliminated, resulting in a clearer image. A zebra-like pattern within the red solid-line frame was formed on the left side of the compensated image since the pixel points of the odd rows were shifted to the right, and the left side was compensated. The width of the zebra pattern represented the number of pixels affected by the inherent distortion of the snake scan.

**Figure 8:**
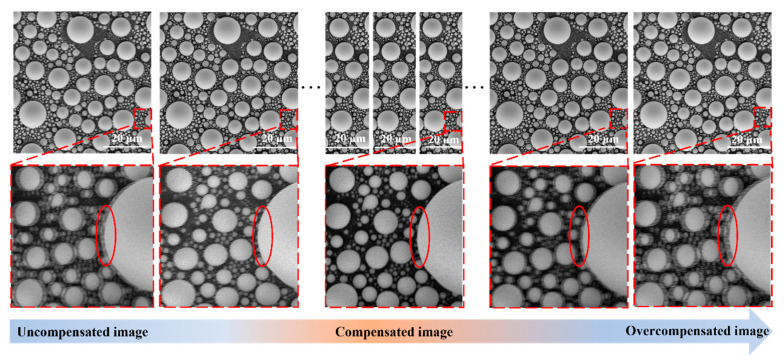
The influence of the change in pixel compensation amount on the image is shown in the enlarged diagram within the red dashed box below (please note the changes within the ellipse).

To demonstrate the importance of the compensation amount in quantifying and correcting image distortion, we experimented with independently controlling the number of compensation pixels. As shown in Figure 8, the uncompensated image showed obvious distortion phenomena such as misalignment and ghosting between the odd and even rows. As the compensation amount gradually increased, ghosting was eliminated, and the image was restored once the distortion compensation reached the optimal level. When the compensation amount became excessive, the number of compensated pixels exceeded the allowable adjustment range, leading to a reappearance of ghosting in the image. The experimental results confirmed inherent distortion and showed that it can be quantified and corrected through pixel adjustments. This distinct periodic misalignment aligns well with the physical mechanism of scanning coil electromagnetic hysteresis and circuit delay, effectively ruling out the dominant contribution of random distortions or feature blurring induced by external factors (e.g., mechanical vibration, electromagnetic interference).

### 2.5. Image Quality Assessment

Four commonly used image quality evaluation indicators were adopted to evaluate the quality of the distortion-corrected images. Firstly, we used the Structural Similarity Index Measure (SSIM) to compare the compensated images with those obtained under raster scanning [34]. This indicator can potentially reflect the alignment effect of the odd and even rows after the snake scan correction, as well as the overall imaging quality. The SSIM ranges from 0 to 1, with values closer to 1 indicating higher structural similarity between the images.

(1) $$SSIM=\frac{\left(2\mu_{x}\mu_{y}+c_{1})(2\sigma_{xy}+c_{2}\right)}{\left(\mu_{x}^{2}+\mu_{y}^{2}+c_{1})(\sigma_{x}^{2}+\sigma_{y}^{2}+c_{2}\right)}$$

where $\mu_{x}$,$\;\mu_{y}$ and ${\;\sigma }_{x}$, $\sigma_{y}$ are the average pixel value and variance of ground truth and predictions, separately, and $\sigma_{xy}$ is their covariance. $c_{1}$ and $c_{2}$ are constants. Edge information is a critical factor in assessing image quality. Images showing clear and well-defined edges are usually of high quality. Then, we employed the Canny edge detection algorithm [32] to extract the edges of the image with the highest SSIM value, thereby further evaluating image quality from the perspective of edge clarity.

Secondly, we used the Natural Image Quality Evaluator (NIQE) to assess images without a reference by comparing local statistical features to a precomputed model of images [35]. A lower NIQE score indicates better image quality. This metric quantifies both the clarity of the microscopic structural details and the uniformity of the grayscale in the SEM image. Finally, spectral analysis [36,37] using Fast Fourier Transform (FFT) is employed to evaluate image distortion through frequency domain characteristics. High-frequency integrity is emphasized, where increased energy in high-frequency components indicates that fine details are better preserved post-correction, with blurring from distortion reduced. Low-frequency uniformity is also focused on, ensuring a smooth distribution of low-frequency components to minimize periodic distortions, such as vertical streaks induced by raster scanning flyback. The correction effects are cross-validated through three independent dimensions-structural similarity, reference-free quality, and frequency characteristics—avoiding accidental results and clearly distinguishing between subjective visual improvements and objective quantitative metric enhancements.

## 3. Results and Discussions

### 3.1. Effect of Scanning Parameters on Distortion

Scanning parameters have a significant impact on imaging quality. First, we uniformly set the SEM acceleration voltage to 20 keV, the electron beam current to 3 nA, and the image size to 1024 × 1024 pixels. The working distance was kept constant at the same magnification, and other parameters, such as focus, were adjusted to their optimal states.

**Figure 9:**
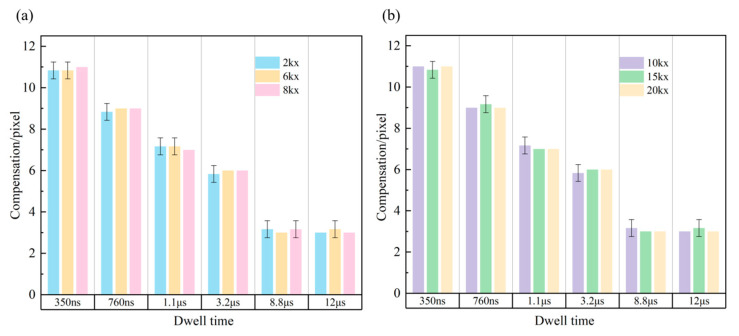
Statistical chart of pixel compensation amounts with dwell times of 350 ns, 760 ns, 1.1 µs, 3.2 µs, 8.8 µs, and 12 µs. (**a**) Magnification of 2kx, 6kx, and 8kx; (**b**) magnification of 10kx, 15kx, and 20kx.

We then used the self-developed external scan controller to control the current change in the scanning coil. Adopting the snake scanning mode, we acquired images with dwell times of 350 ns, 760 ns,1.1 μs, 3.2 μs, 8.8 μs, and 12 μs, at magnifications of 2kx, 6kx, 8kx, 10kx, 15kx, and 20kx, respectively. A statistical analysis of the pixel compensation amount was performed and shown in Figure 9. For example, the pixel compensation amount was 11 pixels when the magnification was 2kx and the dwell time was 350 ns, indicating that the image distortion at this point was 11 pixels. As the dwell time increased, the amount of distortion showed a gradually decreasing trend. When the dwell time reached 8.8 μs, the pixel compensation amount dropped to 3 pixels. However, when we extended the dwell time to 12 μs, the distortion stabilized and remained unchanged. Similar patterns were observed at other magnifications. Once the dwell time surpasses a specific threshold, further increases in dwell time cannot mitigate distortions arising from hardware imperfections or system-inherent limitations. These stem from the scanning system’s hardware constraints, including electromagnetic response delays and digital-to-analog (DA) conversion accuracy bounds. Future research could investigate adaptive compensation algorithms [38], such as machine learning models, to address spatially varying offsets. Certainly, we can also utilize the methods applied by Li L et al. [39] in atomic force microscopy, replacing the triangular wave with B-spline smoothed voltage trajectories to further suppress electromagnetic hysteresis effects at the source. Additionally, if the dwell time remained constant, the amount of distortion compensation for images across various magnifications remained the same. This indicated that, within our study’s parameter range, image distortion was independent of the magnification factor and solely depended on dwell time.

### 3.2. Evaluation of Image Distortion Correction

**Figure 10:**
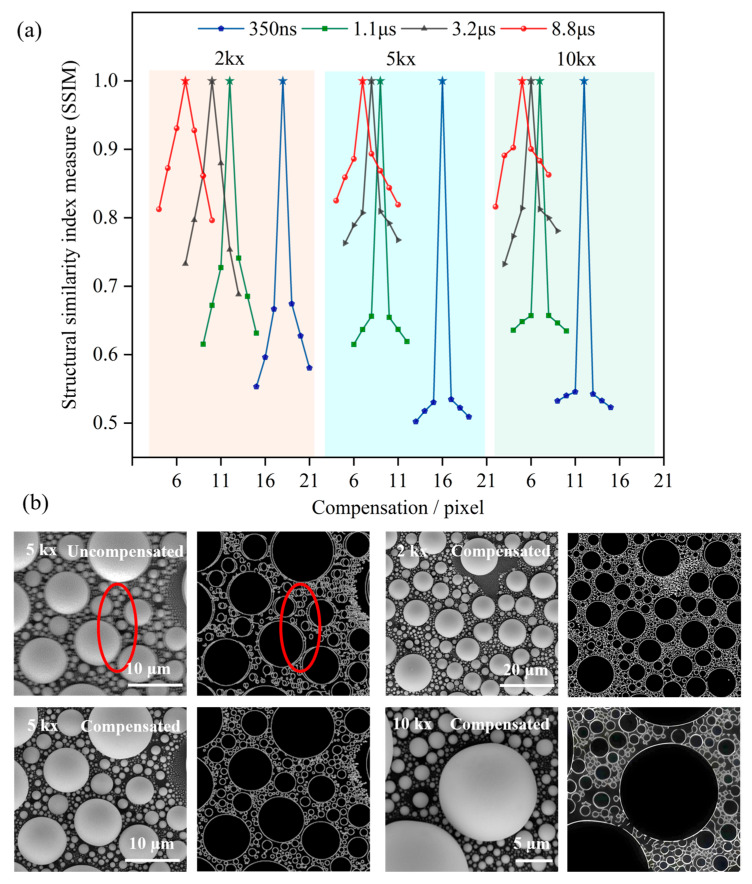
Comparison chart of the SSIM of snake scanning images with different compensation amounts. (**a**) Structural similarity of images with different dwell times at magnifications of 2kx, 5kx, and 10kx (where the stars represent SSIM = 1); (**b**) comparison of the original image and edge extraction (in the red oval shape) at magnifications of 2kx (SSIM = 0.9278), 5kx (SSIM = 0.8935), and 10kx (SSIM = 0.8935).

Figure 10 shows the results of the SSIM quality evaluation analysis after image distortion correction and the comparison after Canny edge extraction. During the SSIM evaluation process, we divided the images into three groups according to the magnification: 2kx, 5kx, and 10kx. For each group, images with dwell times of 350 ns, 1.1 μs, 3.2 μs, and 8.8 μs were collected, respectively, and the image size was set to 1024 × 1024 pixels for all images. Under each dwell time condition, we acquired 6 images with different compensation amounts using snake scanning for comparative analysis. These images cover scenarios of under-compensation, optimal compensation, and over-compensation (Figure 8). Additionally, under the same parameters and sample region, we acquired one reference image using raster scanning.

After acquiring the images, we used the raster scanning images with the same magnification and dwell time as the reference, setting their SSIM value to 1. We then calculated the SSIM values for the images obtained through snake scanning. As shown in Figure 10a, the SSIM values for all snake scanning images (i.e., each polyline) initially increase and then decrease as the compensation amount increases. For instance, for images with a magnification of 2kx and a dwell time of 8.8 μs, the SSIM value gradually rose as the compensation amount increased from 5 to 7. However, the SSIM value began to decline when the compensation amount increased from 8 to 11. This situation was caused by the fact that we had acquired images with under-compensation, optimal compensation, and over-compensation. In the cases of under-compensation and over-compensation, the image quality was poor, and the SSIM values of the images were low. Conversely, in the case of optimal compensation, the SSIM value was highest, indicating that image quality had been effectively improved post-compensation. By comparing the SSIM values of the three groups of images at 2kx, 5kx, and 10kx, we could draw the following conclusions:

(i) Compared to the under-compensated and over-compensated cases, the image distortion compensation strategy adopted in this study was highly effective. For instance, at a dwell time of 8.8 μs, the quality of compensated images closely resembles that of images obtained through raster scanning.

(ii) Since the images we acquired were not subjected to post-processing such as noise reduction, the SSIM values were lower under fast acquisition conditions (dwell time of 350 ns) due to noise interference.

(iii) At the same dwell time, as magnification increases, the SSIM value will decrease slightly. This occurs because, as magnification increases, the image’s details become clearer. Additionally, factors such as noise and distortion have a more pronounced impact on the evaluation of image quality.

We applied the Canny edge detection algorithm to extract the edges of feature points in the images with the highest SSIM values from the 2kx, 5kx, and 10kx groups. As shown in Figure 10b, comparing the edge contours of feature points in the uncompensated and compensated images, it was evident that the edge features of the compensated images were clearer, more continuous, and more regular. Their shapes were closer to the true state. This result demonstrated that the compensation operation could effectively correct image distortion and restore image details, providing strong evidence for objectively evaluating the practical effects of the compensation operation.

**Figure 11:**
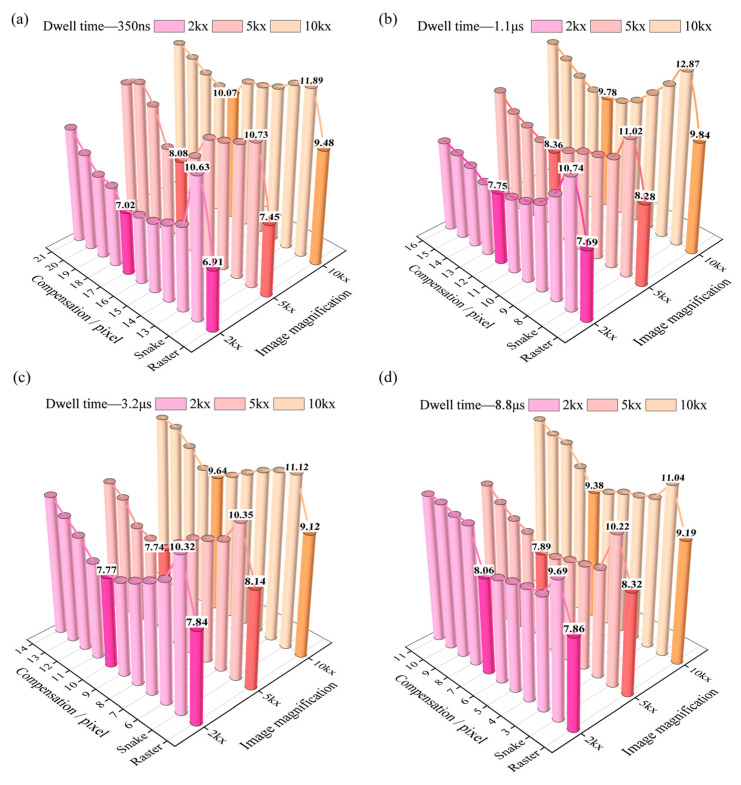
Comparison of raster and snake scanning NIQE at different compensation amounts under magnification of 2kx, 5kx and 10kx. Colors represent different magnification levels, and the optimal compensation in the snake and raster scanning have both been enhanced in color contrast. (**a**–**d**) The dwell time of 350 ns, 1.1 µs, 3.2 µs, and 8.8 µs, respectively.

Figure 11 shows the objective quantitative evaluation of images after distortion correction using NIQE. We divided the images into four groups based on dwell time: 350 ns, 1.1 μs, 3.2 μs, and 8.8 μs. For each group, images with magnifications of 2kx, 5kx, and 10kx were acquired, respectively, and the image size was 2048 × 2048 pixels.

Under each magnification, we used the snake scanning method to acquire 9 images with different compensation amounts and one uncompensated image (labeled “Snake”) for comparative analysis. Among them, the 9 images with different compensation amounts cover different situations, such as under-compensation, optimal compensation, and over-compensation (Figure 8). In addition, under the same parameters and sample area, we used the raster scanning method to acquire one image (labeled “Raster”) for comparison with the optimally compensated image under snake scanning.

We calculated the NIQE values for all the images. The height of each cylinder in the figure intuitively reflects the level of the NIQE evaluation score. A higher score indicates poorer image quality. Among them, we marked the specific scores for the optimally compensated images, the uncompensated images (labeled “Snake”), and the images collected under raster scanning (labeled “Raster”). Figure 11a presents images captured with a dwell time of 350 ns and a magnification of 2kx. As the compensation amount increases from 13 to 17 pixels, the NIQE value gradually decreases. When the compensation amount increased from 17 to 21 pixels, the NIQE value showed an upward trend. Thus, as the compensation amount increased, image quality improved initially, then deteriorated. The uncompensated “Snake” images had the worst quality, while the optimally compensated images demonstrated the best quality, indicating effective improvement through compensation. At the same time, the NIQE score for the raster scanning image was 6.91, while the best-compensated snake scanning image had a score of 7.02. The image quality of the two images was close. Comparing the NIQE values across these four groups, we drew the following conclusions:

(i) Under the same dwell time and magnification conditions, the image quality of snake scanning at optimal compensation was comparable to that of raster scanning.

(ii) At the same dwell time but different magnifications, the compensation amount required to achieve the optimal state was constant.

(iii) The “U”-shaped curve formed by the heights of the bars intuitively reflected the complete process of image quality transitioning from poor to good and then deteriorating again. This also reaffirmed the scenarios of under-compensation, optimal compensation, and over-compensation shown in Figure 8.

The evaluation results and conclusions help us objectively assess the impact of the compensation method on image quality and further demonstrate the feasibility of using snake scanning to quantify image distortion.

### 3.3. Scanning Strategies and Imaging

In the imaging mechanism of raster scanning, there was an extremely short flyback time, resulting in the accumulated distortions forming at the edges of the image. Such twisted or irregular distortions were difficult to quantify. In contrast, snake scanning provided a foundation for quantifying distortion. These distortions can be quantified and compensated through odd–even row splitting, effective feature point registration, and correction algorithms.

We selected images acquired with a dwell time of 3.2 µs and a magnification of 2kx for spectral analysis of both raster and snake scanning. The results from the previous SSIM and NIQE evaluations are summarized in Table 2.

In frequency spectrum analysis, the high-frequency component ratio for raster scanning was 0.1605, slightly higher than 0.1579 for snake scanning. This indicates that raster scanning can display more details or noise to a certain extent. However, high-frequency components were more susceptible to external interference and errors [36]. The broader low-frequency energy distribution range in raster scanning indicates a higher susceptibility to global distortions caused by the flyback mechanism, such as edge warping or bulk displacement. In contrast, snake scanning demonstrates superior stability in large-scale features due to its continuous scanning path, which minimizes inherent scanning-induced artifacts.

Using the raster scanning image as the reference, the structural similarity between snake scanning and raster scanning was as high as 0.8975. According to the NIQE metric, the quality score of snake scanning (7.77) was close to that of raster scanning (7.84), with both meeting natural image quality standards in terms of visual perception. Overall, the two scanning methods demonstrated minimal differences in image quality evaluation. In practical applications, snake scanning can offer a potential direction for the development of adaptive scanning methods in the future.

**Figure 12:**
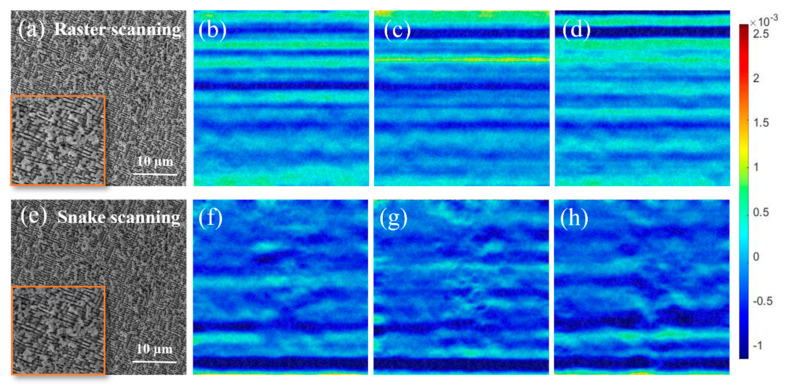
Strain field ($\varepsilon_{yy}$) analysis of continuous pairwise images from a nickel-based superalloy sample, acquired via raster/snake scanning (2048 × 2048 pixels, 5000×, 3.2 μs dwell time) and processed by Ncorr-2D software. (**a**) In the local magnified image within the orange box of raster scanning image, there is no obvious distortion; (**b**–**d**) collect three consecutive images of the area shown in (**a**) and conduct an analysis on them; (**e**) the area in (**a**) was collected using the snake scanning; (**f**–**h**) collect three consecutive images of the area shown in (**e**) and conduct an analysis on them.

Figure 12 shows the strain field in the Y-direction ($\varepsilon_{yy}$) analysis results between undeformed images using commercial Ncorr-2D software (https://www.ncorr.com, accessed on 10 August 2025). This software employs the digital image correlation (DIC) algorithm: by tracking the displacement of speckle patterns between consecutive images, it calculates the strain distribution. Raster and snake scanning modes were employed to capture four consecutive images of the same area of the second-generation nickel-based single crystal superalloy sample. The sample was subjected to mechanical polishing and electropolishing at room temperature, a high-contrast speckle pattern with diameters ranging from 300 to 400 nm (Figure 12a,e). All images had a resolution of 2048 × 2048 pixels, with a magnification of 5000× and a dwell time of 3.2 μs, while other scanning parameters remained consistent.

The strain field maps from raster scanning (Figure 12b–d) exhibit distinct continuous stripes. These artifacts arise from improper incremental deflection of the electron beam in the Y-direction, causing line scan jump defects. In contrast, the strain field maps from snake scanning (Figure 12f–h) show no significant continuous stripes with a notable reduction in strain amplitude, indicating improved stability of the beam stepping in the Y-direction. This observation validates the earlier point that during raster scanning, the voltage varies in a sawtooth pattern with an associated flyback time (Figure 3). Snake scanning leverages the continuity of triangular wave voltage to achieve more stable electron beam stepping in the Y-direction, thereby mitigating vertical jump errors.

As observed in Figure 12f–h, localized color inhomogeneity remains in certain regions. While snake scanning can mitigate electron beam jump errors in the Y-direction, it cannot fully eliminate them. This limitation may stem from additional interfering factors or uneven offsets smaller than our pixel calibration values. To address localized distortions, future work will involve subdividing images into smaller regions and applying nonlinear interpolation compensation. Compared to traditional raster scanning, snake scanning enhances the visibility of horizontal displacements, facilitating the detection of defects occurring in the horizontal scanning direction. For second-generation nickel-based single crystal superalloys, this is crucial for identifying horizontal defects like intergranular slip or dislocation motion along specific crystal planes, which helps in studying material deformation mechanisms and failure modes, thereby enabling targeted corrections and optimizations. The enhanced visibility of horizontal displacements aids in identifying latency issues related to slow electron beam deflection and detector response delays. These insights guide targeted adjustments to scanning parameters and hardware calibration, enabling systematic optimizations.

To further clarify the advantages of the snake scanning-based correction strategy, it is necessary to compare it with the traditional distortion compensation method widely adopted in raster scanning. As discussed previously, raster scanning suffers from irregular edge distortion due to its flyback mechanism, and the conventional solution is “fly-back time” compensation. Traditional fly-back time compensation alleviates local edge distortion in raster scanning via line-end pauses but fails to resolve systematic distortion. Moreover, accumulated pause time compromises imaging efficiency. In contrast, our snake scanning combined with a software correction method maps inherent systematic distortion through odd-even row misalignment, enabling global quantitative correction while enhancing imaging efficiency. The core difference lies in whether systematic distortion can be quantified and resolved while balancing efficiency and applicability.

## 4. Conclusions

In this study, we utilized a self-developed external scan controller and software system, and employed a snake-shaped scanning strategy to quantify and correct the distortions present in the original images. We examined the principles and forms of inherent distortion and their effect on image quality. Distortion was quantified through statistical analysis by evaluating compensated pixels and identifying key influencing parameters. Finally, we addressed the impact of distortion using a unilateral compensation method and compared image quality between raster and snake scanning, confirming the efficacy of the snake scanning method for quantifying distortion. The quantification and compensation of inherent distortion provide a reference for optimizing the design of the scanning coil, improving the electron beam control system, and enhancing the speed and accuracy of the system. Potential applications of this method extend beyond the current research and may play a significant role in other image-processing fields. For example, the snake scanning does not require the flyback process like raster scanning, which can reduce the damage to the sample by the electron beam. Although the current correction method effectively reduces distortion, it still cannot eliminate image distortion. This limitation arises from systematic and random errors during the measurement process, such as fluctuations in electron beam stability and noise interference from the detector. The hardware and methodology currently developed achieve distortion and drift correction [40], with quantization accuracy at the 1-pixel level. We will focus on sub-pixel-level iterative optimization to enhance offset correction in the future.

## Figures and Tables

**Table 1:** Comparison of offsets in the X and Y-directions.

	Offset in the X-Direction							Offset in the Y-Direction				
	3kx	4kx	5kx	6kx	10kx	20kx	3kx	4kx	5kx	6kx	10kx	20kx
350 ns	11	11	11	11	11	11	0	0	0	0	0	0
760 ns	10	10	10	10	10	10	0	0	0	0	0	0
1.1 μs	7	7	6	7	7	6	−1	−1	0	0	0	0
3.2 μs	6	6	6	6	6	6	0	0	−1	0	0	0
8.8 μs	3	3	4	3	3	3	−1	−1	0	0	0	0

**Table 2:** Comparison of imaging quality with different scanning methods.

Image Quality	Spectrum Analysis		SSIM	NIQE
	High-Frequency Proportion	Low-Frequency Distribution Range		
Raster scanning	0.1605	81,896,931,073.8228	1	7.84
Snake scanning	0.1579	81,729,043,840.3511	0.8975	7.77

## Data Availability

The original contributions presented in this study are included in the article/Supplementary Materials. Further inquiries can be directed to the corresponding authors.

## Supplementary Materials

The following supporting information can be downloaded at: https://www.mdpi.com/article/10.3390/ma19010016/s1, Figure S1: The developed SEM external scanning and imaging system [40]. MCU: microcontroller unit. (First Generation); Figure S2: The developed SEM external scanning and imaging system.
