# Supplementary material for: Snake Scanning for SEM: Quantification and Correction of Its Inherent Misalignment Distortion Using an External Scan Controller
Source: Materials (Basel). 2025 Dec 19;19(1):16. doi: 10.3390/ma19010016 (PMC12786985; doi:10.3390/ma19010016)
Supplement: Supplementary file 1 [file materials-19-00016-s001.zip › materials-3995289-supplementary.pdf]

Supplement materials

The PCB includes two waveform generation units, two Bessel filters, a signal acquisition unit, a data-processing unit, and a Gigabit Ethernet transmission interface.

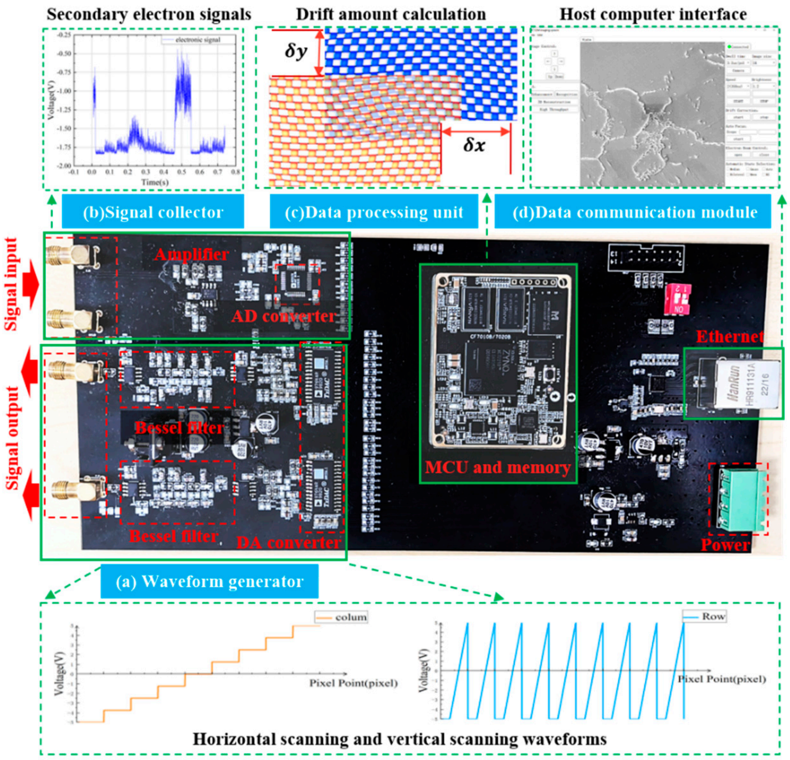

Figure S1 The developed SEM external scanning and imaging system [40]. MCU: microcontroller unit. (First Generation)

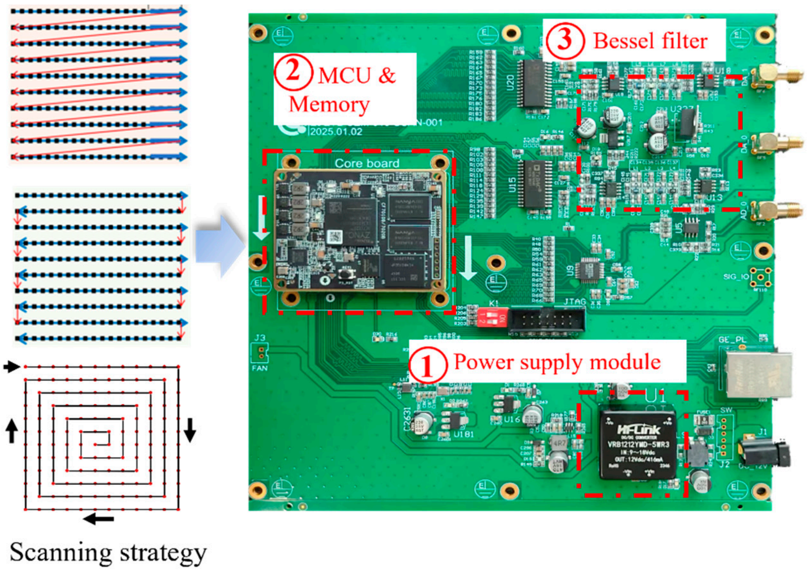

Figure S2 The developed SEM external scanning and imaging system.

(The second generation with high integration in this study)

Waveform generation: The two waveform generation devices consist of two digital-to-analog converters (D/A), two power amplifiers, and two Bessel filters, aiming to output the row and column scanning voltages. The DA conversion chips of the two waveform generation devices both adopt AD9764 (ADI company), with 14-bit resolution. The power amplifiers both adopt OPA690 (TI company) with a bandwidth of 500 megahertz.

The seventh-order active Bessel filter is composed of a power amplifier LM7171 (TI company), capacitors, and inductors, and it has two functions: signal amplification and filtering. The two waveform generation devices can adjust the scanning waveform up to ±12V, with an adjustment accuracy of 0.3mV. The data calculated by the microcontroller unit (MCU) was transformed into the voltage values by using the D/A converter. The voltage was then amplified by the amplifier and filtered by a seventh-order Bessel filter.

Signal acquisition: The signal acquisition device consists of a differential amplifier and an analog-to-digital converter (A/D). The AD conversion chip adopts AD9226 (ADI company), with 12-bit resolution. The differential amplifier uses AD8132 (ADI company) with a bandwidth of 360 megahertz. The differential amplifier has good linearity and excellent noise suppression performance. This device is to capture the secondary electron signal detected by the secondary electron detector and convert it into a digital signal. The signal acquisition device is connected to the signal output end of the secondary electron detector via a signal input interface.

Data processing: The data-processing unit consists of an MCU and a data storage unit. The MCU adopts the field programmable gate array (FPGA) of ZYNQ-7020 (Xilinx company), which integrates a dual-core Advanced RISC Machine Cortex-A9 Multiprocessor Core (ARM Cortex-A9 MPCore) processor. The main frequency rate of ZYNQ-7020 can reach up to 667MHz, endowing it with high computational capabilities. The data storage unit employs DDR4 memory (Micron company) with a data transfer rate of up to 4266 megahertz. The data-processing unit calculates the drift between the reference image I_0 and the real-time image I_t, computes and outputs a waveform signal, corrects image drift, and ensures synchronization between the scanning waveform and the acquisition signal.

Data communication: The data communication module is also known as the upper computer interface. The Gigabit Ethernet data communication module is used to transmit the image matrix generated by the data-processing unit to the host computer for image display and analysis. The data communication module adopts the YT8511C chip (Motorcomm Electronic Technology Co., Ltd), which can provide a maximum data transmission rate of 1000 Mbps.
